# Being Merle: The Molecular Genetic Background of the Canine Merle Mutation

**DOI:** 10.3390/genes11060660

**Published:** 2020-06-17

**Authors:** László Varga, Xénia Lénárt, Petra Zenke, László Orbán, Péter Hudák, Nóra Ninausz, Zsófia Pelles, Antal Szőke

**Affiliations:** 1 Department of Genetics, Microbiology and Biotechnology, Institute of Biological Sciences, Faculty of Agricultural and Environmental Sciences, Szent István University, H-2100 Gödöllő, Hungary; lxenia3@gmail.com (X.L.); nora.ninausz@gmail.com (N.N.); Szoke.Antal@mkk.szie.hu (A.S.); 2 Institute for Farm Animal Gene Conservation, National Centre for Biodiversity and Gene Conservation, H-2100 Gödöllő, Hungary; hudak.peter@hagk.hu; 3 Department of Animal Breeding and Genetics, University of Veterinary Medicine Budapest, H-1078 Budapest, Hungary; zenke.petra@univet.hu (P.Z.); pelles.zs@gmail.com (Z.P.); 4 Department of Animal Sciences, Georgikon Faculty, University of Pannonia, H-8360 Keszthely, Hungary; orban.laszlo@georgikon.hu

**Keywords:** dog, coat color, merle, *PMEL*, short interspersed nuclear element (SINE), splicing, mosaicism

## Abstract

The intensity of the merle pattern is determined by the length of the poly(A) tail of a repeat element which has been inserted into the boundary of intron 10 and exon 11 of the *PMEL17* locus in reverse orientation. This poly(A) tail behaves as a microsatellite, and due to replication slippage, longer and shorter alleles of it might be generated during cell divisions. The length of the poly(A) tail regulates the splicing mechanism. In the case of shorter tails, the removal of intron 10 takes place at the original splicing, resulting in a normal premelanosome protein (PMEL). Longer tails generate larger insertions, forcing splicing to a cryptic splice site, thereby coding for an abnormal PMEL protein, which is unable to form the normal fibrillar matrix of the eumelanosomes. Thus, eumelanin deposition ensuring the dark color formation is reduced. In summary, the longer the poly(A) tail, the lighter the coat color intensity of the melanocytes. These mutations can occur in the somatic cells and the resulting cell clones will shape the merle pattern of the coat. When they take place in the germ line, they occasionally produce offspring with unexpected color variations which are different from those of their parents.

## 1. Introduction

**Figure 1:**
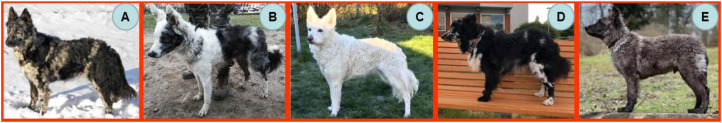
Representative examples for various merle phenotypes of the Mudi breed: (**A**) classic; (**B**) double; (**C**) hidden; (**D**) harlequin; (**E**) dilute.

Several papers have been published recently regarding the molecular genetics of the merle phenotype (Figure 1A–F), including three major articles offering theoretical explanations [1,2,3] and two breed-specific papers [4,5]. The aim of our review is to provide a comprehensive analysis of each of these results and hypotheses, as well as compare them with those of previous papers [6,7] which identify the merle mutation at the molecular level. In addition, we will also provide a detailed review on how this mutation controls the development of various merle phenotypes.

## 2. What Was Known Prior to the Molecular Genetic Identification of the Merle Mutation

Merle is among the most exciting coat color mutations of dogs both phenotypically and genotypically. The autosomal semidominant inheritance of this mutation was described nearly a century ago. Two alleles have been identified and distinguished: the wild “*m*” allele and the mutant “*M*” (or merle) allele [8].

The genotype of dogs exhibiting the typical merle phenotype (Figure 1A) is *M/m*. The presence of the *M* allele results in a fainter/diluted background color and the appearance of fully-colored patches of various sizes and locations, giving a marbling pattern to the body. The merle allele typically modifies the eye color to blue or heterochromatic and that of nose and paws to appear pink-spotted [9].

Although the merle allele segregates in several breeds, there is no breed, where it would be fixed. From these, the Australian Shepherd Dog and Catahoula Leopard Dog are both notable, due to the fact that, in these breeds, the merle is a highly-preferred phenotype [3,7].

Breeders call individuals with the *M/M* genotype as double-merle (Figure 1B). These homozygous dogs exhibit more pronounced phenotypes than the heterozygotes, typically having a lighter background coat color and containing only smaller patches of the original coat color pattern, although some of them are entirely white. Their eye color is predominantly blue, and they also experience frequent ocular (microphthalmia, abnormal iris) and auditory defects, to the extent of being completely blind or deaf [9,10]. In fact, merle to merle matings are officially prohibited by kennel clubs, as they have a 25% probability of producing *M/M* homozygotes [4].

Hidden merles (Figure 1C) also possess the *M* allele, however, phenotypic appearance of the merle pattern is inhibited by the recessive epistasis of mutations in the *MC1R* (melanocortin receptor) locus formerly known as locus “*E*” [11,12,13,14,15,16].

Although cryptic merle individuals do carry specific mutant alleles of the gene, they do not express the classic merle phenotype. The reason for this was discovered later (for details see Section 3.2). These dogs exhibit the merle character in traces only, however among the progeny from a cryptic mating, puppies having the merle pattern are known to appear [6].

The fourth main phenotypic category in merle breeds is harlequin, where the original pigmentation appears in the form of scattered dark spots on a white background. There can be differences in this pattern among breeds; for example, the harlequin Mudi tends to have extended white markings at locations not typically shown in the breed (e.g., chest, collar, tip of extremities), whereas there are minimal markings on the body (Figure 1D) [5]. 

While merle patterning is not considered desirable among breeders of Great Danes, the harlequin phenotype is widely popular. The harlequin (*H*) locus is assumed to be the modifier of the merle gene, specifically in this breed. The hypothesis theorized that the dominant *H* allele produces the harlequin pattern in *H/h* heterozygotes, while the homozygous *H/H* genotypes are thought to be embryonic lethal [17]. Several genes were tested as candidates for the *H* locus, but neither of these were segregating together with the harlequin pattern [13]. Later, the *H* locus was mapped to a 3.27 Mbp region of chromosome 9, also containing the *PSMB7* gene encoding the β2 catalytic subunit of the proteasome. A T>G mutation of exon 2 causing a glycine>valine replacement co-segregated with the harlequin pattern in the case of Great Danes [18,19].

## 3. The Molecular Genetics of Merle Mutation

### 3.1. Identification of the Merle-SINE (Short Interspersed Nuclear Element) Mutation and Its Mode of Action

Linkage analysis was performed on an Australian Shepherd pedigree by using microsatellite markers which resided in close proximity to putative candidate genes for the merle. Genetic analysis of *MITF* (microphthalmia-associated transcription factor), *PAX3* (paired box gene 3), and *SOX10* (SRY-box10) excluded these genes as possible merle loci, however, a new putative gene was mapped to a 5.5 Mbp region of CFA10 [10]. This location was promising, as it showed conserved synteny with HSA12q3 harboring an obvious candidate *SILV* (silver; also called *PMEL17*; premelanosome protein) which plays a fundamental role in premelanosome biogenesis [20,21] and causes hypopigmented phenotypes in different species [22,23,24,25].

Linkage disequilibrium (LD) mapping was performed on nine merle and 32 non-merle Shetland Sheepdog samples with 279 microsatellite markers. The single marker showing significant allelic difference between the two groups was in a region on CFA10 harboring also the *PMEL17* gene. Additional samples, seven merle, two double merle, and eleven non-merle dogs from the same breed confirmed the LD. Sequence analysis identified a single mutation in merle individuals [6]: a tRNA-derived canine-specific repeat, a so-called SINE-Cf (short interspersed nuclear element *Canis familiaris*) inserted into the boundary of intron 10 and exon 11 in reverse orientation [26]. From dogs which were heterozygous for the merle mutation, a ~200 bp and ~500 bp fragment with the SINE insertion could be amplified from exon 11 and the latter fragment segregated together with the merle phenotype [6,7].

This phenomenon and the function of *PMEL17* made it an obvious candidate. This gene encodes the premelanosome protein (PMEL), a major component of the eumelanosomal matrix. PMEL is a pigment-specific protein forming fibrillar sheets on which melanin can be polymerized [27]. It is produced within the endoplasmic reticulum and trafficked to the melanosome [28]. *PMEL* fibrils are necessary for the optimal pigment cell function. Those animals either lacking this protein entirely, or where *PMEL* is less functional due to a mutation, are deemed hypopigmented [27]. Thus, *PMEL* is essential for the production and stabilization of the eumelanin, but not pheomelanin [29], because it is assumed that there is no such internal lamellar structure in the latter [30].

Since the mutation is identical by descent in each dog breed, it can be assumed that it originated from a common ancestor prior to the formation of breeds [2,7].

**Figure 2:**
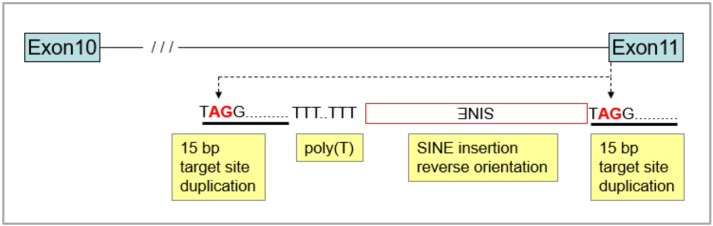
Structure of merle-SINE (short interspersed nuclear element) insertion in the *PMEL* locus. Insertion occurred at the 5′ end of exon 11 in reverse orientation, resulting in target site (red) duplication (15 bp) flanking the merle-SINE element. Sequences are shown as they appear in forward orientation within the *PMEL* locus.

The merle-SINE insertion consists of the following parts: it is flanked on both sides by a 15 bp target site duplication, which is present only at the 5′ end of the exon 11 containing the original splice acceptor site (oSAS). Towards the 3′–5′ direction the next parts compose the head and the body of the SINE, followed by a short dinucleotide (GA) repeat, a cryptic splice acceptor site (cSAS), and finally a poly(T) sequence complementary to the transcribed poly(A) tail [6,7] (Figure 2; from here onwards, poly(A) refers to the mononucleotide tail of merle-SINE, whereas poly(T) labels the complementary mono-T stretch in the genomic sequence).

This structure corresponds to the general structure of SINEs, more specifically to that of the carnivore-specific SINE-Cf family, which constitutes ca. ~8% of the dog genome [31]. This is a relatively recent repeat family which has undergone significant expansion and is still so active that ~7% of the insertion sites are still bimorphic [32,33,34].

SINEs integrated into gene-rich regions can have an influence on gene expression by altering mRNA splicing. The most prominent representatives of SINEs are the primate specific Alu elements that constitute over 10.6% of the human genome and 66% of them are located in the intronic regions [35,36]. If the integration occurred in reverse orientation, the consensus Alu sequence harbors seven potential (i.e., cryptic) 5′ splice donor sites (SDSs) and 12 potential 3′ splice acceptor sites (SASs) [35] and the poly(A) tail in this orientation can serve as a polypyrimidine tract (PPT) required for splicing [37].

SINEs integrated in sense orientation contain three 5′ SDSs and a single 3′ SAS. These splice sites are generally cryptic, thus further mutations are needed to convert them into functional sites. If this does occur, the SINE sequence will be exonized from this site, meaning that it will not be spliced out together with the other intronic sequences and will be included into the spliced mRNA [35]. Exonization is inherently promoted by common characteristics of SINEs; these elements are free from stop-codons and other sequences which are capable of interrupting the open reading frame of the host [38]. 

Splicing is the mechanism which regulates the expression of the merle pattern according to the length of the poly(A) tail. In addition to the splice donor and SAS sequences, there are two additional major structural elements: (i) the branch-point sequence (BPS), which is overall a poorly-conserved element in mammals, with the exception of the central ‘A’ nucleotide [39]; and (ii) the PPT residing between the BPS and SAS [40]. Merle-SINE was inserted in intron 10 and exon 11 boundary into the original splice acceptor site (oSAS), where the cleavage occurs in non-mutant individuals and normal *PMEL* is produced.

**Figure 3:**
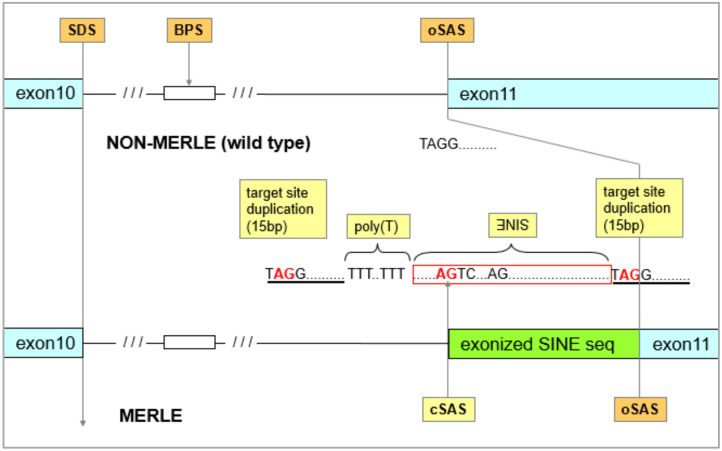
Schematic comparison of the affected region of *PMEL* locus in non-merle (wild type; top) and merle (bottom) dogs. Color codes: exons (light blue); functional elements of the wild type (light brown); new functional elements introduced by merle-SINE (yellow); exonized sequence (green). Abbreviations: SDS—splice donor site; BPS—branch point; oSAS —original splice site; cSAS—cryptic splice site.

BPS is characteristically found at a ~20–50 bp distance from oSAS and once it is extended beyond a certain length, the splicing machinery starts to use alternative cryptic splice sites (cSAS) for the cleavage, although they are less optimal than oSAS [41]. The poly(T) sequence produced by the inverse merle-SINE insertion then plays the role of the PPT in the splicing machinery, enlarging the distance between the BPS and the oSAS. The body of the merle-SINE contains a cSAS sequence which might become functional when the poly-T sequence is ‘pushed too far’ and the splicing machinery starts using the cSAS in a length-dependent manner. When the cleavage occurs at the cSAS, the residual 3′ part of merle-SINE will then be exonized (i.e., translated as a part of exon 11) (Figure 3) [7].

When the alternative cSAS is utilized, a 162 bp fragment of SINE and part of intron 10 are both incorporated into the transcript. Since the reading frame is maintained, 52 additional amino acids become incorporated into the PMEL protein. The merle-SINE exonization occurs near the transmembrane domain (TMD) [1,7].

The longer the poly(T) sequence, the more often cSAS is used for the cleavage resulting in fully, or partially abnormal PMEL proteins. Thus, as the proportion of cSAS/oSAS usage increases, there will be a higher level of fibrillar matrix reduction in the eumelanosomes, as abnormal PMEL has a negative effect on melanocyte viability [1]. This results in fainter color of the mutant animals, explaining the base coat color dilution in the merle pattern.

### 3.2. How is the Length of the Poly(T) Sequence Able to Generate Diverse Merle Phenotypes?

The poly(T) sequence of the merle-SINE is, for all practical purposes, a mononucleotide microsatellite [42,43]. SINEs might play an important role in the evolution of microsatellites, since it is assumed that microsatellite birth is capable of occurring within transposable elements (i.e., SINEs, LINEs, Long Interspersed Nuclear Elements) and the poly(A) tail promotes this process [44,45]. In the human genome, more than 99% of the mononucleotide microsatellites are homopolymer A repeats. The genomic location of these A-rich repeats supports this hypothesis, as they occur most frequently at the 3′ end of LINE-1 or Alu retrotransposons [46,47,48].

The high variability of microsatellites is due to their inherent mutational behavior: the synthesized strand separates from the template strand and may re-associate out of register, resulting in a decreased or increased repeat length during DNA replication. This replication slippage is most pronounced at the mononucleotide repeats. This phenomenon makes it problematic to determine exactly the number of repetitions, since during the PCR reaction—used for the genotyping of microsatellites—the same slippage can take place [49,50].

A LINE-1 transgenic mouse was generated as a model for the purpose of following the early life of the poly(A) tail mononucleotide repeat-length variation dynamics within an individual (somatic) as well as between generations (germ line). Alleles with a poly(A) sequence longer than 100 bp were frequently shortened (13.3–14.8%) generating numerous novel alleles both in somatic and germ cell lineages. In contrast, in alleles with <60 bp length, shortening occurred with low frequency, producing only few allelic variants. Comparison of different tissues was unsuccessful in revealing differences in this aspect. It was shown that longer poly(A) microsatellites might become rapidly shortened within only a few cell divisions following birth, due to replication slippage. On the other hand, elongation was not reported in this study [48].

When orthologous microsatellite repeat-containing regions were compared, dog sequences showed a higher pure-to-unpure ratio for the repeats than 42 other carnivore species. It was assumed that the level of replication slippage is elevated throughout the entire genome of dogs, generating new microsatellite alleles after birth. Accumulating point mutations will gradually disrupt their purity and thus, in principle, suppress the occurrence of replication slippage. If a mutation still occurs, the elevated slippage mutation rate of the canine genome will remove this imperfection by the copy-paste nature of this process and will ultimately purify the microsatellite repeat. Tandem repeats within the coding sequences can contribute to the phenotypic variation. Thus, it is possible that the “slippery-nature” of the dog genome might contribute to the outstanding morphological variability of the species on the whole [51].

As the poly(A) tail of merle-SINE is longer and purer than those of other SINE-Cf sequences, it is more prone to expansions and contractions, including larger ones potentially explaining dramatic shifts in the extent of merle phenotype between generations [1].

#### 3.2.1. Other SINE Mutations in the Dog Genome

There are several additional identified SINE mutations within the dog genome. From the aspect of coat color genetics, a well-known example is the SINE insertion in the agouti signaling protein (*ASIP*; A-locus in classical nomenclature) [14,52]. Another morphological mutation was determined while performing the genetic mapping of the body size of the dogs. A SINE mutation and a single nucleotide polymorphism (SNP) was identified in the second intron of the insulin-like growth factor 1 (*IGF1*) gene in small breeds, while these mutations tended to be absent from those dog breeds having larger body sizes [53,54].

SINE insertions cause several genetic diseases; examples include: (i) a SINE in the hypocretin (orexin) receptor 2 gene causing canine narcolepsy [39]; (ii) a SINE in the protein tyrosine phosphatase-like, member A (*PTPLA*) resulting in gene centronuclear myopathy [55]; and (iii) an intronic SINE insertion in the *FAM161A* gene causing progressive retinal atrophy (*PRA*) in dogs [41].

#### 3.2.2. *PMEL* Mutations in Other Species

The silver (si) mutation in mice results in their premature graying due to the loss of follicular melanocytes [22]. Molecular studies revealed that a single-nucleotide insertion leads to a premature stop codon and loss of the final 25 amino acids from the C-terminal cytosolic domain, resulting in a truncated protein [22,23].

Three color mutations in chicken are related to the *PMEL17* locus. The “dominant white” allele is associated with a 9 bp insertion in exon 10, adding three amino acids into the transmembrane domain. The “smoky” allele also carries this insertion and an adjacently-positioned 12 bp deletion in exon 6. The “dun” allele causes the elimination of five amino acids from the transmembrane domain [25].

The *Z* locus responsible for the silver coat color of horses was mapped on chromosome 6. This missense mutation occurs in exon 11, changing the second amino acid from arginine to cysteine (Arg618Cys) [24].

A three-nucleotide deletion causes the elimination of a leucine in a highly conserved position of the PMEL signal peptide within dun Highland and dun Galloway cattle. This mutation is inherited in a semidominant manner and alters their color in a dose-dependent manner (i.e., heterozygotes (dun) are pale while mutant homozygotes (silver dun) become even more pale than the wild homozygotes) [56].

## 4. Correlating the Merle Genotype with the Phenotype

In recent studies [1,2,3], the length of the poly(A) sequence was determined by automated high-resolution fluorescent fragment analysis from several individuals of different breeds. The resulting genotypes were correlated to the phenotypes that were sorted into several subcategories. These correlations suffer from two primary issues:

1. Phenotype categories were set up by breeders prior to the molecular genetic identification of the merle mutation. As such, these categories might differ substantially from each other by country, breeds or kennel clubs, since judges at competitions might perform the phenotyping based on different considerations. Thus, the basis of phenotyping (i.e., the rate of merle coloration) would be very difficult to standardize globally, even if pictures were available for all animals.
2. Determination of the poly(T) sequence has a certain error rate, which tends to increase slightly with the length of the repeat. Overall precision of the genotyping results can be improved using several technical replicates [1].

**Figure 4:**
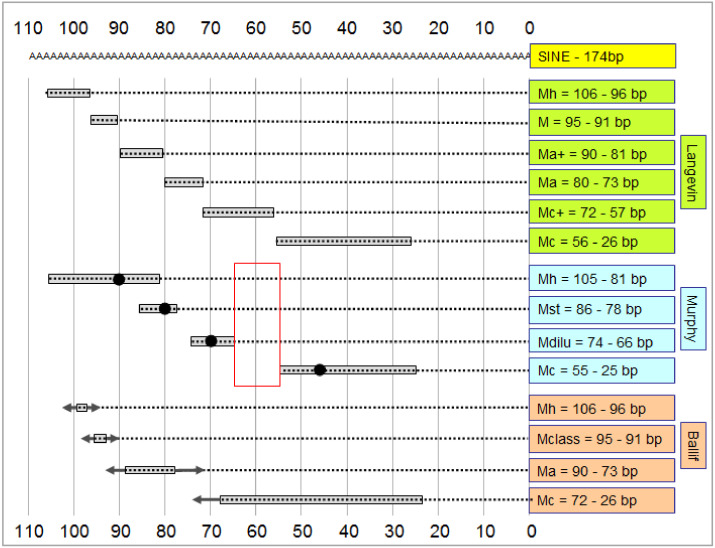
Comparison of the merle-SINE tail lengths of the phenotypic categories using standardized data from prior studies (see [1,2,3]). Numbers on both the top and bottom indicate the length in base pairs. The grey box shows the phenotypic range. The black dot is the most frequent value within the range in the categories presented by Murphy et al. [1]. The red rectangle denotes the range of 55–66 bp above which alternative splicing can occur. The arrows on the two sides of the ranges determined by Ballif and colleagues [2] show the minimal and the maximal extensions of the categories in addition to their overlaps with the neighboring categories. Phenotypic categories in alphabetic order: M—merle; Ma—atypical; Ma+—atypical+; Mclass—classical; Mc—cryptic; Mc+—cryptic+; Mdilu—dilute [1]; Mh—harlequin; Mst—standard. (Note that ‘dilute merle’ of Murphy et al. [1] is different from the diluted (*D*, melanophilin gene, *MLPH*) locus which was identified earlier as one of the classical coat color loci of the dog possessing two alleles—a dominant non dilute ‘*D*’ allele and a recessive ‘*d*’ allele—which in homozygous configuration results in lighter, diluted eumelanistic pigmentation [57,58]).

These recent publications measured the length of the merle-SINE using various scales. Some used the whole length of merle-SINE, whereas others the poly(T) length only. We recalculated their data in its entirety and subsequently standardized them based on the poly(A) tail length, allowing for direct comparisons (Figure 4). The resulting sequences ranged between ~25 bp and ~105 bp in size and each poly(T) length was present in at least one publication, meaning that there were no length ranges without merle alleles in the unified data set.

Langevin et al. [3] distinguished six phenotypic categories and assigned to them six perfectly-joined merle-SINE genotypic ranges (Figure 4). The authors examined 181 individuals belonging to 14 breeds. Genotyping was performed on an ABI 3500 genomic analyzer and the authors declared that this equipment ensured precise quantification of genotypic ranges [3].

Murphy et al. [1] examined 259 dogs belonging to seven breeds divided into four merle categories (Figure 4): cryptic (Mc: 19 individuals), dilute (Md: 18) (Figure 1D), standard (Mst: 161), harlequin (Mh: 41) and non-merle (12, not shown). In some cases, the allelic ranges were overlapping, in others, they were not connected and there was even an interval into which none of the genotypes fell (Mst-Mdilu; Mdilu-Mc) (Figure 4). Separation of PCR products was performed by an ABI 3730xl Genetic Analyzer [1].

Ballif and colleagues [2] also set up four phenotypic classes: harlequin (the longest), classic, atypical, and the shortest cryptic categories. Phenotypic ranges make overlapping transition zones, in which any samples of the neighboring ranges may also be included. They examined 175 animals from three breeds. Separation of PCR products was performed by the ABI SeqStudio Genetic Analyzer with a single base-pair resolution (Figure 4) [2].

From our point of view, the rigid category range system used by Langevin et al. [3] with the above-mentioned resolution appears unlikely, as such minimal changes in the poly(T) sequence are not supposed to cause such drastic shifts between phenotypic categories. The overlapping category ranges in the system of Ballif et al. appear more plausible, as these authors recognized significant genotypic–phenotypic discrepancies even within samples having the same poly(A) length [2].

## 5. Merle at the Cellular Level

During the early stages of embryogenesis, germ cells are separated from somatic cells. Mutations occurring during the successive cell divisions in somites are transmitted to the derived cells in the clone. The size of the clone is proportional to the timing of mutations (i.e., the earlier the mutation takes place during the ontogenesis, the larger the proportion of the mutant sector). Somatic mutations are not inherited between generations. On the contrary, germ line mutations may be potentially inherited, meaning that a certain mutant merle-SINE allele may be transmitted to the progeny by chance, when the gamete carrying this mutation will take part in the formation of the zygote.

### 5.1. Somatic Mosaicism of Merle Mutants

#### 5.1.1. Assumptions Regarding the Development of Merle Coat Color Patterns—Somatic Mutations in the Melanocytes

**Figure 5:**
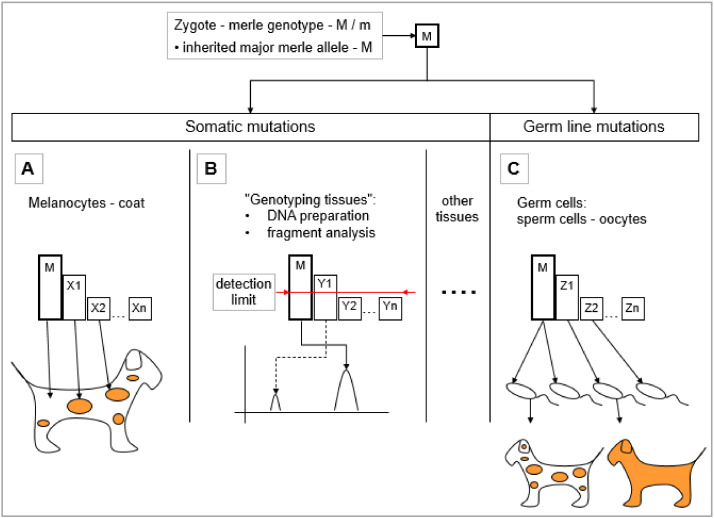
The fate of merle allele (*M*) mutations in the somatic and germ cells. The sketch shows a spotted, heterozygous individual having a mutant, standard merle allele (*M*) and a wild allele (*m*). The standard merle allele is capable of both extension and contraction. An inherited, single merle mutant allele (*M*) may mutate further within the different somatic tissues and within the germ line. In these tissues, the largest cell population will carry the major inherited *M* allele and according to the high mutation rate of the poly(T) sequence, smaller cell populations containing different minor mutant merle alleles, will also exist. The mutational spectra and the proportions of the mutant sector within the various tissues do differ and thus, are denoted here using different letters. (**A**) Melanocytes (mutations X1-Xn); (**B**) “genotyping tissues” (for details see Section 5.1.2; mutations Y1-Yn); and (**C**) germ line (mutations Z1-Zn) are shown. (**A**) In the case of melanocytes, the major mutant cell population will determine the overall coat color, while the different minor mutant cell subpopulations will result in the development of individual patches. (**B**) When we perform genotyping from the DNA of blood lymphocytes, the mutant spectrum structure will be similar. In addition to the major cell population containing the *M* allele, the largest mutant cell population(s) will be detected depending on the resolution of the genotyping platform. Smaller mutant cell subpopulations under the detection limit remain invisible. (**C**) The mutant spectrum of the germ line might also contain mutant cell subpopulations, but not necessarily the same ones as in other tissues. If we analyze these cell populations as a “genotyping tissue” with a genetic analyzer, we will observe a similar spectrum to that shown in (**B**). However, if we analyze several progenies of this individual, we will evaluate a non-representative sample which will not depict the full mutant spectrum of the germ cells.

During embryonic development, melanoblasts migrate away from the neural crest to find their destination, where they differentiate into pigment-producing melanocytes [59,60,61]. The majority of those melanocytes which are carrying the original major inherited mutant allele (*M* allele, see Figure 5) may or may not mutate further during the course of this migration. These melanocytes will ultimately determine the overall color of the coat. PMEL17 protein is produced in the melanocytes [27] and acts to develop the individual merle pattern of the coat if it contains a merle-SINE insertion with a poly(A) tail of a certain length. During these divisions, cells with a merle allele may mutate further and the derived minor cell populations with varying poly(T) sequence length will inherit the new mutant merle alleles. Patches may develop from these types of merle-SINE insertions having contracted poly(T) sequences on the background color. Note that other coat color loci might have an effect on the spotting, for example the spotting (*S*) locus: *MITF* [6,49,62]. According to the current concept, a spot will be lighter insomuch as the melanocytes contain an expanded poly(T) sequence and darker in the event of a shorter one (Figure 5A, X1-Xn). Thus, it is these distinct and separate mutant cell populations which shape the merle pattern. The size of the different patches is proportional to the earliness of the developmental stage when the mutation arises; the earlier it is, the larger the size of the spot.

Murphy and colleagues formed a hypothesis on the relationship between lighter background color and the poly(T) sequence, noting that the length of poly(T) in the cryptic merles is so short that the splicing mechanism is able to use the oSAS exclusively, and hence there is no alternative splicing. As the name suggests, the diluted merle color is a bit lighter compared to the normal background color, since here, the poly(T) sequence extends into the 55–66 bp range. According to the authors’ hypothesis (Figure 8 in [1]), that is the threshold above which alternative splicing occurs, resulting in the production of both alternative and normal transcripts depending on the length of the poly(T) sequence. This proportion might be 25% cSAS to 75% oSAS in the case of dilute merle and 50%/50% for standard merle dogs. In the latter case, the proportion of alternative transcripts is higher which dilutes the base color even further. The figure indicates that splicing will shift to ~100% cSAS usage for the harlequin merle, which leads to the exclusive production of abnormal PMEL and consequently to lack of pigmentation in the background color [1,7].

How then are spots of full pigmentation able to arise in standard/classic and harlequin merle dogs? It is assumed, that somatic reversion can occur in some of the melanocytes of these phenotype categories with longer poly(T) sequences. This results in such a sharp contraction of this repeat sequence, that it is shortened to such an extent that it reaches the ranges of dilute or cryptic merle categories. This permits exclusive usage of the oSAS leading to full pigmentation intensity in these patches [1].

This phenomenon is known as coat variegation, when sectors consisting of mutant cells also appear. Contrary to the standard/classic and harlequin dogs, this variegation was not present in the diluted and cryptic merle categories. Theoretically, replication slippage may result in either expansion or contraction [50], however, a disproportional contraction bias was reported in the examined somatic tissues [1,2].

#### 5.1.2. Somatic Mutations Resulting Mosaicism in the ‘Genotyping Tissues’

At this point, we must introduce the term ‘genotyping tissue’ as a new, simplified category into this particular review. These are tissues which are routinely used for DNA preparation in dogs (e.g., blood and buccal swab). Although sperm is not a typical ‘genotyping tissue’, it should be considered as such from this aspect (see also Section 5.2). Expression of *PMEL* has not been detected in these tissues thus far. In these ‘genotyping tissues’, several cell populations are capable of coexisting in parallel carrying merle-SINE poly(T) sequences of different length. The genotyping procedure to determine the allelic length of these mutations is based on fragment length analysis through capillary electrophoresis. In accordance with the detection threshold of a particular genetic analyzer, an individual peak may be considered either as a bona fide allele or an artifact. Consequently, the inherited major allele as well as mutations occurring in the early stages of differentiation will be detected, however, those of lower proportions might remain undetected due to technological limitations (see e.g., Figure 5B, Y1-Yn).

Murphy and colleagues have not detected larger expansions in these tissues. According to them, there can be several explanations for this phenomenon: Firstly, the longer the poly(T) sequence, the lower its amplification efficiency. Therefore, it is plausible that the expansion does occur, but it remains undetectable with the fragment analysis procedure used. Secondly, expansion may have a negative effect on melanocyte survival or their division and this may be the cause of the contraction predominance [1].

This unequivocal contraction dominance serves as a sound basis for comparison of the coat (with melanocytes) and the ‘genotyping tissues’. The latter showed contraction for 45% of the standard and harlequin merles, while each of these dogs had at least a few spots with full color intensity observable in their coats. At the same time, it is interesting to note that the contraction rate detected in the ‘genotyping tissues’ was in rough correlation with the proportion of full pigmentation of the individuals. A worthwhile further aim would be to isolate melanocytes from skin tissues and then compare the poly(T) length with the intensity of the pigmentation [1].

Ballif and colleagues detected both expansions and contractions, although substantially more from the latter. Among the 175 individuals examined, 24 showed *m/M/M* and two exhibited *M/M/M* mosaicism. In addition to the major merle allele, these three dogs falling into the cryptic range carried some expanded minor alleles as well. There were two expansions and three contractions among the atypical animals. Individuals in the classic and harlequin phenotype categories had a major allele characteristic for their category and also some minor alleles, with the exception of a dog with a classic phenotype. This individual carried both an expanded and a contracted minor allele in addition to the major one [2].

Langevin et al., found mosaicism having three or more alleles in 30 animals (16.6%) out of the 181 merles tested. Major and minor alleles were defined on the basis of peak height in the fragment analysis. Allele contractions were detected for 27 dogs. Expansions were shown in three animals; however, the authors considered these more to be technical artifacts than real expansions. Based on these results, they suggested that length extension does not exist at all in the case of the merle-SINE [3].

In our study performed on Mudis, 22 out of 123 merle individuals turned out to be mosaic: 20 with contractions and two with expansions [5]. This result is in agreement with the biased proportions as described by others, demonstrating the existence of poly(T) expansion at the merle-SINE insertion [1,2].

### 5.2. Effect of the Germ Line Mosaicism on the Merle Pattern

Although the expression of PMEL was not shown in germ cells, presence of the merle-SINE can still be detected. Replication slippage of the poly(T) sequence, as described above, is capable of occurring during cell divisions within the germ line too, thus expanded and contracted poly(T) length alleles can appear. In this way, germ cells with varying allelic versions may coexist at the same time. This phenomenon is known as germ cell mosaicism.

Several new allelic versions may arise from the inherited major merle allele within the germ line as well. The question is: Which germ cells, from the several million present, will take part in the actual zygote formations from which the offspring of the litter will develop? In principle, it should be proportional to the ratio of the germ cell populations carrying either the major or deviating mutant alleles. Any given litter, however, cannot be considered to be a representative sample of all possible germ cell varieties of a given pair of parents (Figure 5C, Z1-Zn).

Germ line mosaicism can be examined by several different approaches:

1. Germ cell volume: Germ cells may be examined in mass, as one sample in a volume as a ‘genotyping tissue’. Practically speaking, this denotes a DNA preparation from a sperm volume, as this approach does not work with oocytes. Fragment analysis can be performed to determine the major allele in addition to those mutant alleles which arose from cell divisions during the early stages. Since alleles that represent smaller proportions of the whole mutant cell population may remain under the detection limit, this analysis provides only a partial picture of the germ line mosaicism.
2. Individual sperm typing: By genotyping a high number of individual germ cells for the length of the merle-SINE insertion [63], a more precise estimate can be obtained regarding the level of germ line mosaicism, individual mutations, and their actual proportion. However, this approach is suitable only for research purposes, and is not practical for the testing of individual families, because it is both expensive and labor-intensive.
3. Mutant spectra comparison from ‘genotyping tissues’ within the family: The genotyping of that parent carrying the *M* mutation along with the offspring from ‘genotyping tissue’ is an indirect way for deducing the inherited major allele in every single progeny, and thus, assessing the derived mutant alleles. If the fragment analysis of ‘genotyping tissue’ identifies a merle-SINE insertion having the same poly(T) size to be the major allele typically with the highest peak intensity in the parent-offspring pair, then the progeny has inherited the paternal, non-mutant allele in the germ line. However, if the original parental *M* allele is not present in the offspring and it possesses a longer or shorter allele instead, then it must be a germ line mutation, insomuch that the parentage is indeed certain. The recent, aforementioned articles applied this approach for the study of germ line mosaicism [1,2,3].

Murphy and colleagues identified five de novo expansions and seven contractions that occurred during gametogenesis. Since they examined only heterozygous dogs carrying a single copy of the merle-SINE mutation, their results demonstrate that these length mutations are not the products of unequal crossovers during meiotic cell divisions. It is interesting that nine of these mutations were inherited from the sire, substantiating the observation that the mutation rate is higher in spermatogenesis than in oogenesis [1].

Ballif et al., demonstrated using several family materials supplemented by photographs as well as the chromatograms of the fragment analysis, that both expansions and contractions occur in the germ line [2].

Langevin and colleagues suggested that parental merle alleles are conserved in length between generations and according to them it is highly probable that the merle-SINE expansion does not exist. In their view, the practical importance of this hypothesis is that breeders need not worry about the mating of cryptic merle individuals with wild homozygotes, because no unexpected merle puppies are able to be born to such litters, as the cryptic allele cannot mutate to an expanded allele with a longer poly(T) sequence in the germ line [3].

Their explanation for the unexpected occurrence of merle puppies (unanticipated on the basis of the parents’ merle-SINE genotypes) is that the parental genotypes were determined from the ‘genotyping tissues’ and not from germ cells. Sperm cells contain many minor merle alleles which do not necessarily match to the mutation spectra of the different ‘genotyping tissues’ and any of them may potentially take part in the fertilization; thus, this discrepancy offers an explanation for the appearance of unexpected genotypes and phenotypes among the progeny. The authors recommend the testing of sperm cells derived from the sire for possible mosaicism [3].

The question is whether expansion can occur in the germ line, or not. Articles presenting argument supporting the possibility of expansion have demonstrated their evidence using family materials showing parent–offspring comparisons, and as such, this hypothesis seems more widely supported. ‘Genotyping tissues’ seem to be suitable for the detection of the original, inherited parental mutant *M* allele. These demonstrate multiple examples of not only contractions, but also of expansions, where the parental *M* allele is not present in the progeny, but a new mutant allele with increased length appears as a new major allele instead. At the same time shorter, contracted alleles are also visible in addition to the major allele on the chromatogram of the fragment analysis, however these must be somatic mutations generated in the ‘genotyping tissues’ and have nothing to do with the germ line. These authors point out the significant bias in the ratio between the overrepresented contraction over the lesser-represented expansion [1,2]. This asymmetry fits the general behavior of mononucleotide repeats, and as such the proportion expressed in this biased ratio can be legitimately expected [48].

All in all, there seems to be sufficient proof pointing to the occurrence of merle-SINE expansion in the germ line as well. Consequently, the possibility of unexpected merle offspring appearing in litters from cryptic merle to homozygous wild-type matings does exist. Theoretically, the single sperm typing approach [63] might be able to accurately estimate the germ line mosaic status of a cryptic sire. However, in practice, it is not suitable for implementation to routinely genotype a representative number of sperm cells. Using an ejaculate as a ‘genotyping tissue’ will not offer a perfect solution either, despite being better suited than those containing somatic cells, as it detects germ line-based genotypes. This approach will only reveal the most likely offspring genotypes to be transmitted by the sire, but not those rare variants whose proportion is below the detection threshold of fragment analysis. Moreover, since sperm cells are continuously produced in the testes, this genotype pattern might change during the lifetime of the male.

## 6. Conclusions

According to our knowledge at present, the prerequisite of a merle coat pattern in dogs is the presence of the merle-SINE insertion in the *PMEL* gene. The length of the merle-SINE poly(A) tail regulates the merle pattern ranging from the invisible cryptic merles to the harlequin phenotype. However, other genetic factors as well as the environment are also deemed to exert additional effects on this trait. This is why a specific merle-SINE poly(A) length is capable of producing a wide range of phenotypes. It should be noted that typing of this sequence with a single base-pair resolution might be challenging from a technical standpoint. A single base-pair difference might not result in a visible phenotype category shift, and so, in our view, it would be more realistic to apply overlapping genotypic ranges in accordance with the phenotypic categories. Additionally, evidence shows that larger sample sizes do not result in better phenotype-genotype correlations. The overall picture might be clearer with the additional testing of other color loci. Moreover, single-cell (melanocyte, germ cell) genotyping might lead to better understanding of merle mosaicism.

As the majority of recent publications on this topic concluded, both large contractions and expansions can occur in the poly(A) tail of merle-SINE between generations. Although the proportion of expansions is lower than that of contractions, it must not be considered as merely a technical artifact. They are capable of occurring both in the somatic tissues and in the germ line. These extensions result in the occasional unexpected appearance of merle phenotypes in the litters of parents that are phenotypically non-merle, but genotypically cryptic merle. It is for this reason, that merle-SINE genotyping is just as important in these cryptic crosses as in the case of hidden merle matings.
